# Infection Inspection: using the power of citizen science for image-based prediction of antibiotic resistance in *Escherichia coli* treated with ciprofloxacin

**DOI:** 10.1038/s41598-024-69341-3

**Published:** 2024-08-22

**Authors:** Alison Farrar, Conor Feehily, Piers Turner, Alexander Zagajewski, Stelios Chatzimichail, Derrick Crook, Monique Andersson, Sarah Oakley, Lucinda Barrett, Hafez El Sayyed, Philip W. Fowler, Christoffer Nellåker, Achillefs N. Kapanidis, Nicole Stoesser

**Affiliations:** 1 Department of Physics, University of Oxford, Parks Road, Oxford, OX1 3PU UK; 2 Kavli Institute for Nanoscience Discovery, University of Oxford, South Parks Road, Oxford, OX1 3QU UK; 3 School of Infection and Immunity, University of Glasgow, Glasgow, G12 8TA UK; 4 Department of Microbiology and Infectious Diseases, Oxford University Hospitals NHS Foundation Trust, Oxford, OX3 9DU UK; 5 National Institute of Health Research Oxford Biomedical Research Centre, John Radcliffe Hospital, Oxford, OX3 9DU UK; 6 Nuffield Department of Women’s and Reproductive Health, Big Data Institute, University of Oxford, Oxford, OX3 7LF UK; 7 Nuffield Department of Medicine, University of Oxford, John Radcliffe Hospital, OX3 9DU Oxford, UK

**Keywords:** Antimicrobial resistance, Bacteriology, Bacterial infection, Translational research, Fluorescence imaging

## Abstract

Antibiotic resistance is an urgent global health challenge, necessitating rapid diagnostic tools to combat its threat. This study uses citizen science and image feature analysis to profile the cellular features associated with antibiotic resistance in *Escherichia coli*. Between February and April 2023, we conducted the Infection Inspection project, in which 5273 volunteers made 1,045,199 classifications of single-cell images from five *E. coli* strains, labelling them as antibiotic-sensitive or antibiotic-resistant based on their response to the antibiotic ciprofloxacin. User accuracy in image classification reached 66.8 ± 0.1%, lower than our deep learning model's performance at 75.3 ± 0.4%, but both users and the model were more accurate when classifying cells treated at a concentration greater than the strain’s own minimum inhibitory concentration. We used the users’ classifications to elucidate which visual features influence classification decisions, most importantly the degree of DNA compaction and heterogeneity. We paired our classification data with an image feature analysis which showed that most of the incorrect classifications happened when cellular features varied from the expected response. This understanding informs ongoing efforts to enhance the robustness of our diagnostic methodology. Infection Inspection is another demonstration of the potential for public participation in research, specifically increasing public awareness of antibiotic resistance.

## Introduction

Antibiotic resistance is an escalating global health concern, necessitating the development of new technologies such as rapid tests for antibiotic-resistant bacteria to mitigate its impact. Rapid identification of which bacterial species is causing an infection and its resistance profile has been shown to both optimize antibiotic use and enhance patient outcomes^1,2^. Currently, typical diagnostic tests rely on time-consuming bacterial culture growth, taking a minimum of 12–48 h to produce results. Alternative rapid tests focus on identifying resistance-associated genes, but these may not always directly correlate with phenotypic resistance^3–5^. Antibiotic resistance poses a significant threat to individual and public health by potentially rendering common antibiotics ineffective in treating bacterial infections, but public awareness of the use of antibiotics and the impact of antibiotic resistance remains incomplete^6^.

Citizen science is a research method that integrates public outreach with data collection, typically with members of the public collecting or analysing data related to the natural world as part of a collaborative project with professional scientists^7,8^. Citizen science collaborations between volunteers, often called citizen scientists, and research teams can play an important role in educating the public about scientific concepts and have been instrumental in recognizing complex patterns within biological data, starting with research in ecology and extending to various biological fields, including protein folding, DNA sequence alignment, electron microscopy, and microbiology^9–11^. These projects enable individuals of diverse backgrounds and expertise levels to contribute to scientific data collection and analysis, empowering them to actively advance and acquire knowledge in various disciplines. Public involvement broadens the spectrum of available data, perspectives, and ideas, leading to more comprehensive and innovative research outcomes. Successful examples, such as the Great Backyard Bird Count and the Merlin Bird ID app^12,13^, demonstrate citizen science's potential to enable large-scale data collection and analysis, raise awareness, inspire future scientists, and promote environmental and civic responsibility.

The public can be effectively engaged in citizen science projects using various strategies, including hosting events, utilizing social media platforms, partnering with educational institutions and community organizations, and offering training and educational resources, but most of these approaches engage only 10s–100s of individuals. Online platforms which simplify access for large-scale, global public engagement in targeted or diverse citizen science projects include Zooniverse^14,15^, SciStarter^16^, and Foldit^17^ amongst others^18^. For participants, Zooniverse offers a unique and engaging way to learn about science, participate in real research, and connect with like-minded individuals^19^. Zooniverse is currently the largest and most popular citizen science platform, with more than 1 million volunteers^14^.

A previous project hosted on Zooniverse, Bash the Bug^11^, successfully engaged citizen scientists to look at images of bacterial growth and identify their resistance to antimicrobial drugs. This demonstrated how citizen science can be used for antimicrobial resistance research and the development of novel diagnostic tools. We were interested in what insights could be gained from a citizen science collaboration with volunteers providing human interpretations of our datasets, which consist of thousands of micrographs of antibiotic-sensitive and antibiotic-resistant *Escherichia coli* cells. We are developing a diagnostic method that relies on a microfluidic device for the direct capture and identification of bacteria^20^ and associated antibiotic resistance from clinical samples using microscopy, with the goal of providing a result in less than an hour by conducting the test on cells from the clinical sample, eliminating lengthy culturing steps. We recently developed a deep-learning model which can classify individual *E. coli* cells as ciprofloxacin-sensitive or resistant with 80% accuracy (which results in high-confidence classifications of populations of bacteria) based on morphological changes to the sub-cellular structure^21^. This approach could provide direct information about a strain’s antibiotic susceptibility and the heterogeneity of response within a population, which is lost in genotype-based assays.

The continued development of these single-cell, imaging-based classification methods requires learning about bacterial heterogeneity, and an understanding of why certain cells within a sample are misclassified is essential. We therefore developed a project on Zooniverse called Infection Inspection to leverage the power of citizen scientists towards optimising our novel method, and to engage the public in an antibiotic resistance-focused project. We first trained volunteers to recognize cellular phenotypes associated with ciprofloxacin-sensitive and ciprofloxacin-resistant *E. coli*, and then used their classifications to learn which features facilitate accurate classification, and which lead to ambiguity and misclassifications. Our aim was to use their classifications and misclassifications to see if humans were able to identify more nuanced features than our machine, to make our machine learning-based classifier more robust to atypical phenotypes, whilst simultaneously educating the citizen scientists about antibiotic resistance. We found that our machine learning model picks up a greater breadth of features, but by investigating the common features of images, we identified the key features necessary for *E. coli* to be classified as ciprofloxacin-sensitive or resistant.

## Methods

### Image dataset

The project dataset was made up of 49,074 individual images of ciprofloxacin-treated *E. coli* cells generated for previous work^21^ (Table 1). All bacteria had been chemically fixed and stained using 4′,6-diamidino-2-phenylindole (DAPI) as the nucleic acid stain and Nile Red as the membrane stain. The initial dataset was composed of 11,074 256 × 256 Red–Green–Blue (RGB) images of *E. coli* cells from 5 clinical strains (EC1, EC2, EC3, EC5, and EC6, reported previously^21^), with clinical strains defined as ciprofloxacin-resistant (minimum inhibitory concentration [MIC] > 0.5 mg/L) or ciprofloxacin sensitive (MIC ≤ 0.25 mg/L) using European Union Council on Antimicrobial Susceptibility Testing (EUCAST) breakpoints^22^. The minimum inhibitory concentration is a standard metric of antimicrobial resistance and is the lowest concentration of antibiotic that inhibits growth of bacteria in an overnight culture^23^. The MICs of the strains were calculated empirically by E-test strip (Liofilchem), or, where the MIC exceeded the maximum range of the strip, by a 1:1.5 broth dilution of the antibiotic^21^. In the initial dataset, all strains were treated at 10 mg/L ciprofloxacin for 30 min; this concentration, well in excess of the EUCAST breakpoint, was chosen to induce an antibiotic response within the short, 30-min treatment period. EC4 was excluded because its MIC is equal to the treatment concentration, and we define the expected phenotype based on whether the treatment concentration was greater than or less than the MIC (see “Accuracy and participation analysis”). A second dataset of 38,000 images included two of the *E. coli* strains (EC1 and EC3) treated at 9 concentrations ranging above and below their MICs (from 16 to 0.001 mg/L ciprofloxacin) for 30 min.

**Table 1:** *Escherichia coli* clinical isolates used for the Infection Inspection project.

Strain	Year	Cip S/R/I	Cip MIC (mg/L)	Number of images	Correct/total classifications	ZN accuracy	Model accuracy
EC1	2016	S	0.008	15,900	187,729/280,469	66.9 ± 0.2%	84.9 ± 0.6%
EC2	2015	S	0.03	3883	41,013/53,251	77.0 ± 0.4%	81.6 ± 1.2%
EC3	2018	I	0.5	21,529	238,057/377,612	63.0 ± 0.2%	71.0 ± 0.6%
EC5	2017	R	8	2882	41,732/45,599	91.5 ± 0.3%	86.2 ± 1.3%
EC6	2014	R	72	4877	42,650/68,575	62.2 ± 0.4%	76.3 ± 1.2%

Strains are listed, along with their year of collection; ciprofloxacin susceptible, resistant, or intermediate (Cip S/R/I); ciprofloxacin MIC (mg/L); number of images; correct classifications and total classifications; Zooniverse user accuracy (95% confidence interval); and model accuracy (95% confidence interval). All strains are *Escherichia coli* isolated from bloodstream infections in the United Kingdom. EC1 and EC3 have many more images than the other strains because we included images from samples treated at multiple antibiotic titrations (see Fig. 2b).

All bacteria were imaged in an automated workflow as agarose-mounted samples in phosphate buffered saline (PBS) on a Nanoimager-S fluorescence microscope (Oxford Nanoimaging) using the multiple acquisition capability of the microscope with autofocusing on each field of view. The image segmentation for background removal was done with an optimised model of Mask-RCNN adapted from a standard implementation^21,24,25^.

### Development of infection inspection with the Zooniverse project builder workflow

Infection Inspection was designed as a citizen science project on the Zooniverse platform^14^ using the Project Builder^26^, a free-to-use web browser application enabling research teams to build and contribute projects to the site. During the building process, we developed an initial workflow, tutorial, and project field guide. Datasets were added to the project as .png images using the Subject Set upload tool within the Zooniverse Project Builder.

Infection Inspection was submitted for internal review in August 2022 and went to beta reviewers in September 2022. We wanted volunteers to classify our images of *E. coli* as “Sensitive” or “Resistant.” We encouraged users to make their best classification, even if they were unsure (Supplemental Methods: Infection Inspection Tutorial). A label of “Image Processing Error” was added to account for a small number of images where a problem in the imaging pipeline made the image impossible to classify, such as a membrane with no DNA, DNA and membrane channel mismatch, or a segmentation error (Supplemental Methods: Infection Inspection Tutorial). In response to beta feedback, we improved our project terminology and added explanations to the field guide and instructions for how to classify ambiguous or unusual cells (Supplemental Methods: Infection Inspection Field Guide).

During the beta test, we noticed that user accuracy did not improve with the number of classifications done. To help users learn from their own misclassifications, we implemented user feedback for a set of 30 tutorial images. These images had a ground truth classification of “Sensitive,” “Resistant,” or “Image Processing Error” and users would receive feedback on their accuracy immediately after submitting a classification for one of these images. Tutorial images were shown to users with decreasing probability: 0.5 for the first 5–10 images, declining to 0.25 by 20 images, and 0.05 after 50 images. The retirement limit was set to 20, meaning that each image was considered complete once it was classified by 20 unique volunteers.

The Zooniverse platform allows anyone to volunteer for projects and does not require any specialized training or qualifications beyond the project’s tutorial and Field Guide (Supplemental Methods). The tutorial and field guides were written in line with guidance on communicating with the public on antibiotic resistance from the Wellcome Trust^27^. We solicited and implemented feedback from non-experts, public engagement experts, and a secondary school biology teacher on the language used in the project before submitting for beta testing.

### Accuracy and participation analysis

On completion of the project on 10th May 2023, the project data file which included all classifications was downloaded in .csv format from the Zooniverse site. Only classifications performed from go-live (7th Feb 2023 17:40 UTC) to full dataset completion (10th May 2023 21:40 UTC) were included in the analysis. Image identifiers were matched back to the original strain and metadata including known MIC, treatment concentration, clinical antibiotic susceptibility phenotype, and predicted classifications were assigned to each data point. The predicted classification was defined as the expected response of the strain to the antibiotic. If the MIC was less than the treatment concentration, the expected phenotype was sensitive. If the MIC was greater than the treatment concentration, the expected phenotype was resistant. For instance, if the MIC for a particular strain was 0.03 mg/L and the treatment concentration was 10 mg/L, that strain was categorized as sensitive. Conversely, if the MIC was 72 mg/L and the treatment concentration was 10 mg/L, the strain was labelled as resistant. All usernames were anonymised to ‘User_1, _2, etc.’. For the accuracy determination, any classifications of images that were part of the training/feedback dataset or classifications of “Image processing error” were removed. Summary statistics were performed in R (version 4.2.3) using the R package vegan^28^ (version 2.6-4) and plotted with ggplot2^29^ (version 3.4.4). Accuracy was graded as whether the user’s classification matched the image’s predicted classification as defined above. We report accuracies with the 95% confidence interval of the mean, by the Wald method with continuity correction.

We used the Gini coefficient to characterise the extent of inequality in the distribution of classifications by volunteers. The Gini coefficient derives from a metric for income inequality^30^ and has been applied to measure inequality in volunteer contributions previously^19^. We calculated the Gini coefficient with the following formula:

$$1 - \left( {2 \times \frac{{\mathop {\sum {(proportion\;of\;total\;classifications)} }\nolimits_{{}}^{{}} }}{total\;users}} \right).$$

### Image feature analysis

CellProfiler^31,32^ (version 4.2.6) was used to extract image features from the dataset. The RGB .tif images were split into grayscale single-color images using the ColorToGray module. Then, the IdentifyPrimaryObjects module was used with default settings to identify the Membrane object from the red channel. For the Nucleoid object, two-class Otsu thresholding was used with default settings because it segmented diffuse nucleoid regions more accurately. Intensity measurements for each object were measured using the MeasureObjectIntensity module. Size and shape measurements were extracted using the MeasureObjectSizeShape module. All measurement data were exported using CellProfiler to an SQLite database^33^ and selected measurements were converted to .csv files with DB Browser for SQLite^34^ (version 3.12.2).

Further image feature analysis was completed in Python and R scripts, available at https://github.com/KapanidisLab/infection_inspection. Images were excluded from analysis if more than half of their classifications were “Image Processing Error.” An accuracy threshold of 0.5 was chosen to compare images that were most frequently classified correctly or incorrectly. For example, if a cell’s predicted classification was Sensitive, based on its MIC and the treatment concentration, and it was classified as Sensitive by more than 50% of users, it would be labelled as Correct Sensitive. A cell from the same strain and treatment condition that was classified as Resistant by more than 50% of users would be labelled as Incorrect Sensitive. This yielded four sets of images whose features could be compared: Correct Sensitive, Correct Resistant, Incorrect Sensitive, and Incorrect Resistant. Images were called Most Correct if they were classified correctly with a ratio greater than 0.94, corresponding to roughly 19 correct classifications of 20. By categorising images based on the consensus of the users’ classifications, we minimised the impact of misclassifications by individual users.

For feature comparisons between groups of cells displaying ciprofloxacin-resistant or sensitive phenotypes, we performed two-sided *t*-tests with Bonferroni corrections for multiple comparisons using the ggpubr^35^ (version 0.6.0) and Rstatix^36^ (version 0.7.2) packages. We compared the distributions of the values associated with cellular phenotypic features to a normally distributed Random Noise feature generated by numpy.random.normal^37^. A principal component analysis with 2 principal components was performed using the 7 measured image features and the Scikit-learn PCA function^38^. Before analysis, all feature measurements were normalised with Standard Scaler from Scikit-learn^38^. For the dataset with multiple ciprofloxacin concentrations, the principal component analysis with 2 principal components was performed in R with the prcomp^39^ function from the stats library (version 4.1.3) and plotted with ggplot2^29^ (version 3.4.3).

Independently, we extracted the feature importance values from a Random Forest classifier with 100 trees and a minimum of 3 samples per leaf that had been trained using Scikit-learn^38^ on images that were randomly allocated to a 75–25 train-test split and then scaled with Standard Scaler. The Random Forest classifier was evaluated by cross-validation by the Mean Absolute Error and was then applied to the test dataset to make predictions.

For each image, we calculated SHAP (SHapley Additive exPlanation) values for each feature using Kernel SHAP, a model agnostic implementation for Python^40^. This method, which derives from game theory approaches, measures an importance value for each feature for each image classification, and has been shown to correspond well to intuitive human feature impact estimates**.**

### Ethics approval

This work was reviewed by the University of Oxford’s Research Governance, Ethics and Assurance team (Oxford, UK). The bacterial cells imaged and shared for classification were obtained as part of a separate study with ethical approval (London—Queens Square Research Ethics Committee, REC ref: 17/LO/1420), analysed anonymously as part of this project and thereby not subject to the Department of Health’s *UK Policy Framework for Health and Social Care Research* (2017), and not requiring further sponsorship, research ethics review, nor Health Research Authority (HRA) approval. Citizen scientists who contributed classifications to the Infection Inspection project were deemed contributors and not research participants.

## Results

### The Infection Inspection project engaged a large cohort of users

**Figure 1:**
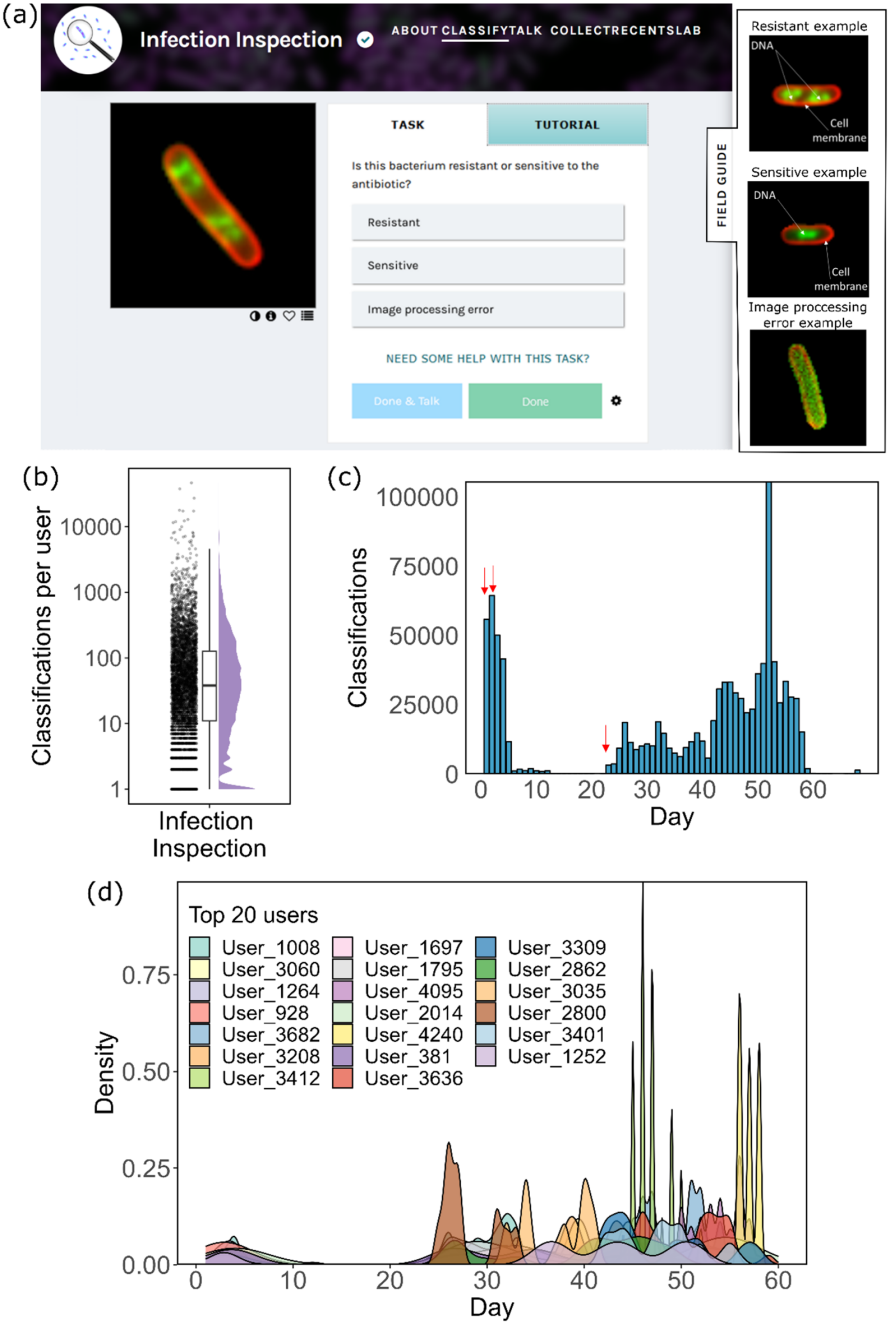
Engagement of users with Infection Inspection. (**a**) Task page for users of Infection Inspection. Users were presented with an image of a bacterial cell and were asked to select from one of three options to classify the image. Accessing the field guide provided examples of each cell type as exampled in the subset panel. (**b**) Distribution of the number of classifications by user (n = 5273 users, n = 1,045,199 classifications). Each dot represents an individual user that performed a classification on at least 1 non-training set image. The box represents the middle 50% (IQR) of the users and the mid-line indicates the median number of classifications. (**c**) The distribution of classifications performed on each day of the project. Red arrows indicate the time of each data batch upload. (**d**) Density mapping of activity for each of the top 20 users (by number of classifications) over the course of the project highlighting the differences in patterns of contribution. Day 1 on the x-axis represents the first day that the user engaged with the project.

Infection Inspection was launched on the Zooniverse platform on the 7th of February 2023 (Fig. 1a) and was promoted via multiple platforms, including webpages, print magazines, in-person outreach events, and emails for Zooniverse users. An initial dataset comprising 30 training and 5000 test images was available to users and was completed within just 18 h. A second dataset of 6074 images was subsequently uploaded and was completed in 72 h; then a final dataset of 38,000 images was uploaded and was completed in 35 days (840 h). A total of 5273 unique users performed at least one image classification and overall, 1,045,199 classifications were made between the project launch date and May 10th, 2023. After removing classifications of the training dataset, a total of 4927 users remained, covering 1,003,588 classifications (Fig. 1b). The median number of classifications performed by users was 38, however the variation in number of classifications per user was large, with 56 users performing > 2000 individual classifications. The maximum classifications undertaken by any given user was 46,289.

Engagement with the project correlated with the upload of new data, with spikes in classifications occurring within 1–2 days after upload (Fig. 1c). Among the 20 most engaged users, return to the project was common, with these users returning to the project on several occasions throughout (Fig. 1d). The Gini coefficient for our user participation was 0.81, which means that the most prolific volunteers contributed a large proportion of our project’s classifications, or our project attracted many casual users, or both. The Gini coefficient for Infection Inspection is close to the mean Gini coefficients of the most popular ecology (0.80), astronomy (0.82), and transcription (0.81) projects on Zooniverse and higher than the average Biomed project score of 0.67 based on a previous analysis^19^.

### Volunteers classified *E. coli* cellular phenotypes with accuracy comparable to deep learning

**Figure 2:**
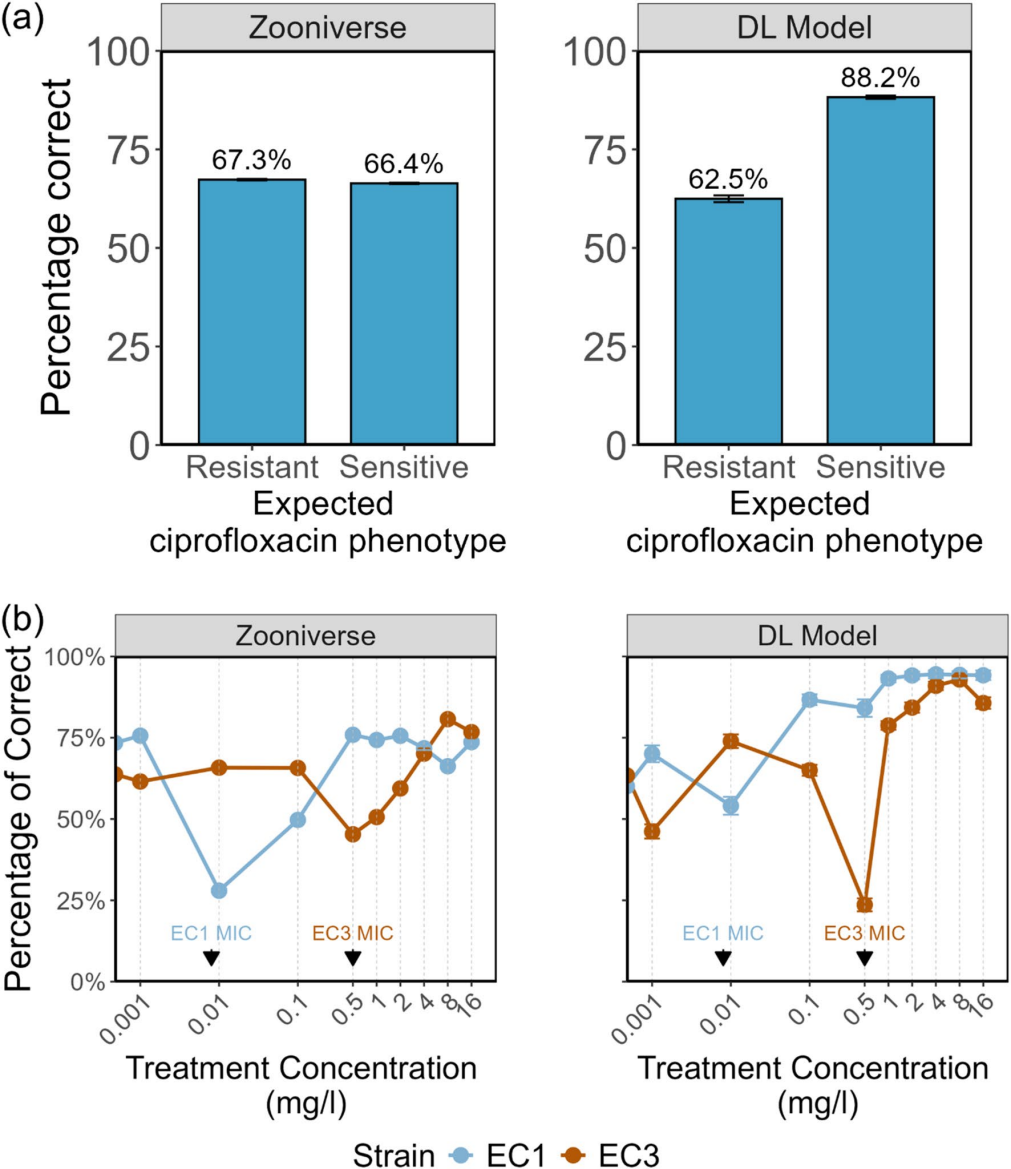
Classifications of images by Infection Inspection users. (**a**) An aggregated plot of how accurate Zooniverse users or the model were at categorising an image into either the resistant or sensitive category based on the expected phenotype for the given sample. Error bars indicate the 95% confidence interval. DL model denotes the classifications of the deep learning model. (**b**) The line plots visualise the percentage of images that were correctly categorised as either resistant or sensitive as expected based on the treatment concentration, by the users or the model. Error bars indicate the 95% confidence interval. Each subplot shows the data for a different *E. coli* strain with the treatment concentration on the x-axis. The known, predetermined MIC for each strain is indicated on the plot using arrows.

We assessed the accuracy of user classifications in distinguishing bacteria as either ciprofloxacin-resistant or sensitive, based on the ciprofloxacin treatment concentration relative to the Minimum Inhibitory Concentration (MIC) for each strain. When we aggregated the data from all three dataset uploads, users achieved an accuracy of 66.4 ± 0.2% in classifying sensitive cells (Fig. 2a). The accuracy for classifying resistant cells was similar, standing at 67.3 ± 0.2% (Fig. 2a). We also employed the same images to test a deep-learning model^21^. Compared to the volunteers, the model was less accurate in classifying resistant cells (62.5 ± 0.9%; Fig. 2a), but more accurate in classifying sensitive cells (88.2 ± 0.4%).

Given that the number of classifications performed by users varied, we examined whether there was a correlation between accuracy and the total number of images classified. Despite having ten users who classified > 10,000 images, we observed no significant relationship between accuracy and the total number of images classified (Fig. S1a). Additionally, the duration of user activity on the project did not influence classification accuracy (Fig. S1b).

### Classification accuracy depended on the antibiotic concentration used for treatment

In the third dataset uploaded, we introduced cells from an experiment in which two strains (EC1 and EC3) were treated with varying concentrations of ciprofloxacin to see how the proportion of cells classified as resistant is related to the MIC of the strain. Some of the concentrations used were below the MIC, and thus we expected to see no significant phenotypic changes; on the other hand, some of the concentrations were above the MIC, and should produce phenotypic changes. These treatments allowed us to investigate whether both users and our deep learning model could detect changes in cell structure based on a graduated treatment concentration. For one of the strains (EC1), users correctly classified the cellular changes close to 75% of the time for all treatment concentrations except the one closest to the known MIC of the strain (Fig. 2b). At this specific treatment concentration (0.01 mg/L), accuracy in identifying the response dropped to nearly 25%. A similar trend was observed in the model's predictions. While accuracy was highest at treatment concentrations of 0.1 mg/L and above, the greatest confusion was encountered when the treatment concentration approached the MIC of the strain (Fig. 2b). This pattern of increased confusion was also observed for the second strain (EC3), which had a different MIC (0.5 mg/L).

### Differences in DNA morphology led to the most confusion in correctly classifying images

**Figure 3:**
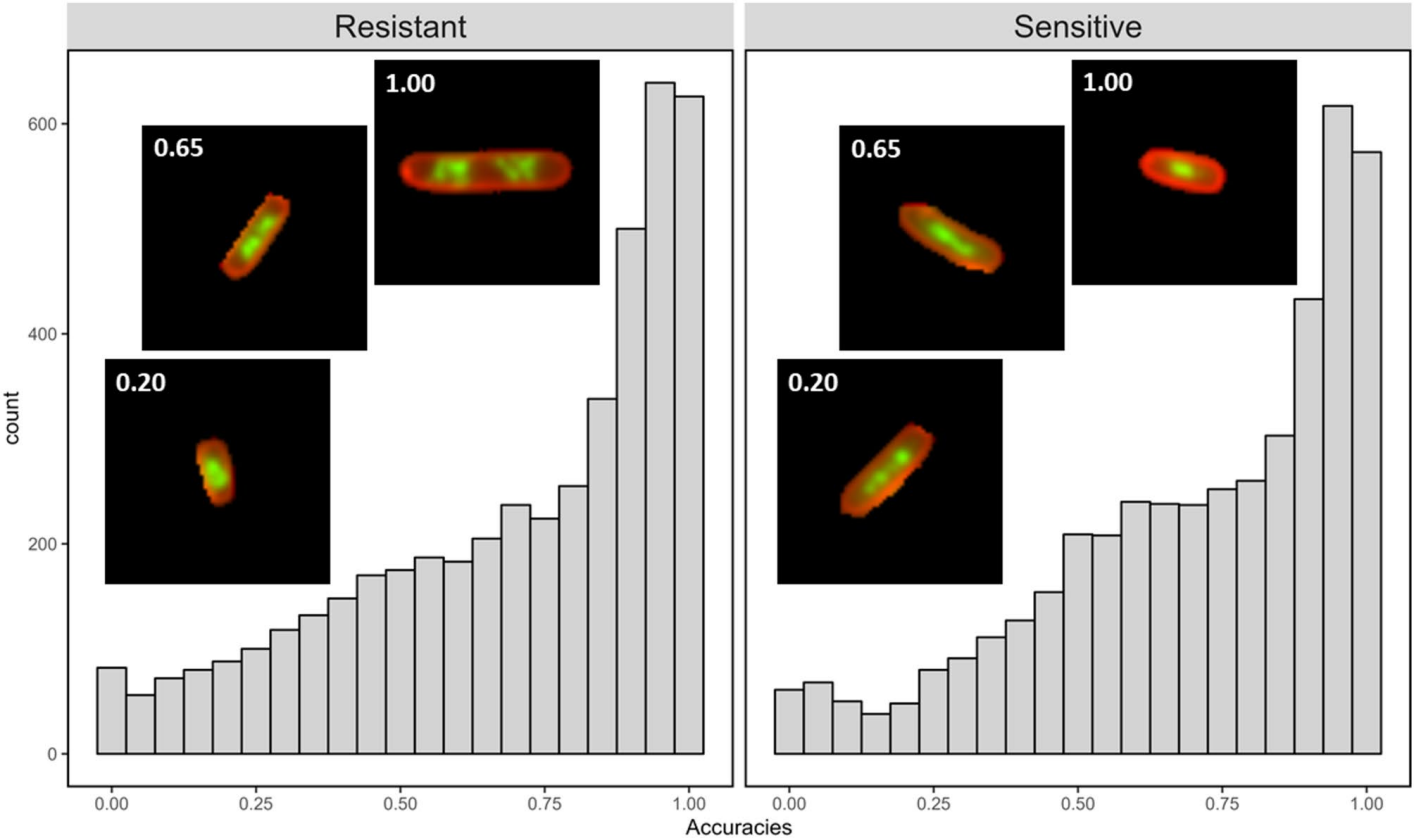
User accuracy varied by image for both resistant and sensitive cells. Histograms of the user accuracy on images of *E. coli* treated at 10 mg/L for resistant and sensitive cells. Representative images of resistant and sensitive cells with low classification accuracy (0.20), intermediate classification accuracy (0.65) and high accuracy (≥ 0.95) are shown. Both resistant and sensitive cells show a skew, with many cells being classified correctly nearly always and some almost never. However, both populations also have many ambiguous images that were classified correctly by around half of the users.

Some images were more frequently misclassified than others. In the first uploaded dataset of ciprofloxacin-sensitive and ciprofloxacin-resistant cells treated at 10 mg/L, the classification accuracy histograms are skewed, with many images almost always classified correctly, and others almost never (Fig. 3). This suggested that, while many cells displayed the expected cellular phenotype when exposed to ciprofloxacin, there were sub-populations with atypical features.

For this specific analysis, images were assigned to the “Incorrect” class if they were classified with less than 50% accuracy (1755 images), and otherwise to the “Correct” class (7074 images). Cells were excluded from the image feature analysis if they were labelled as an “Image Processing Error” by more than half of the users who classified the image; this removed 230 images.

To explore why some cells were more frequently misclassified than others, human-interpretable image features were measured with CellProfiler^31,32^. Seven features were chosen for their potential biological relevance to the ciprofloxacin response (Fig. S2). To characterize the compaction, heterogeneity, and quantity of DNA, we measured the number of DNA regions per cell, the mean and standard deviation of the integrated intensity of the DNA regions, the mean standard deviation of the DNA intensity, and the area fraction occupied by the nucleoid regions. The cell shape was described by the form factor of the membrane and the major axis length was used to measure the cell size.

**Figure 4:**
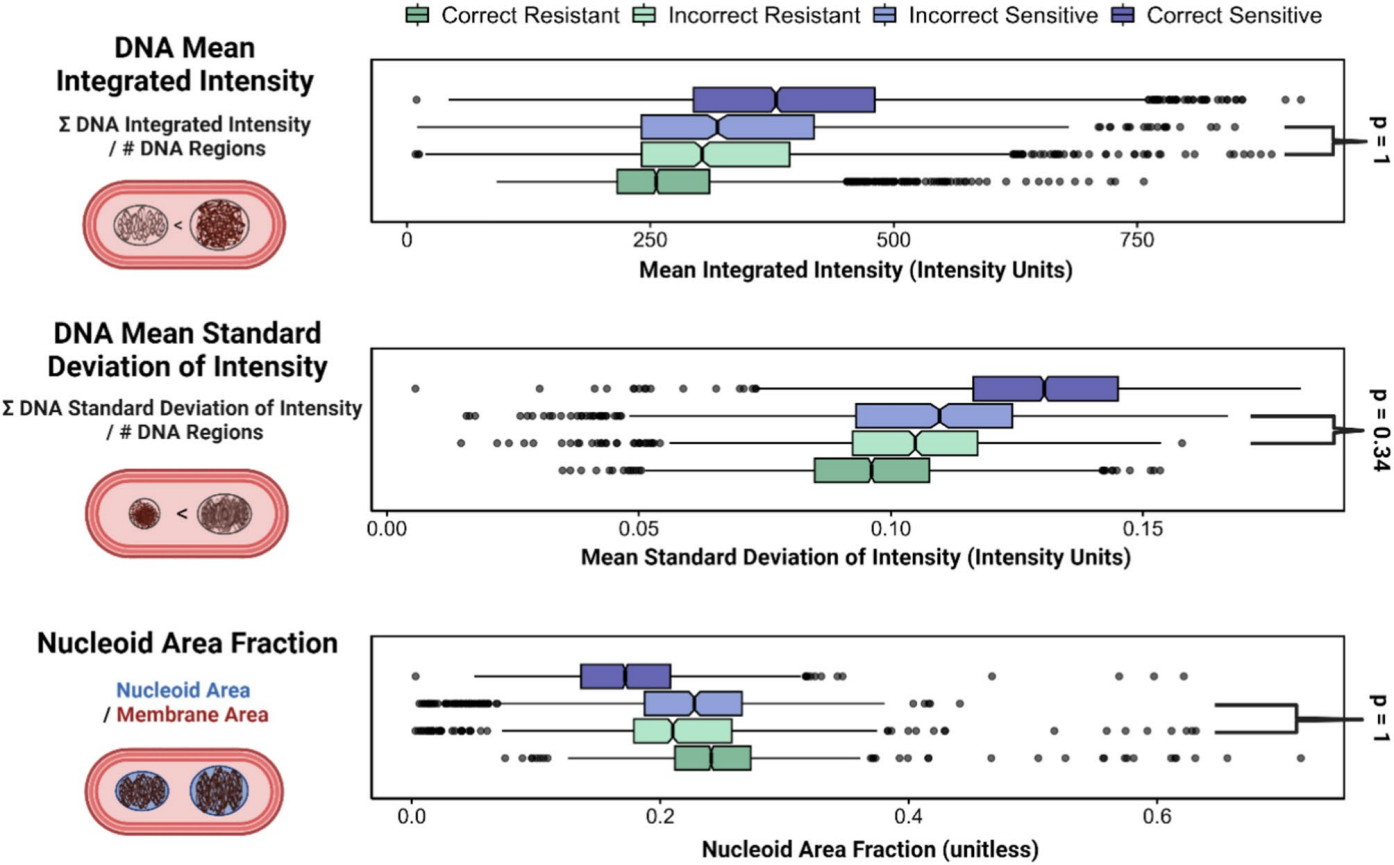
Cells that were classified incorrectly have more similar features. For three image features related to DNA heterogeneity and compaction, illustrations and definitions of which are shown on the left, the Incorrect Resistant and Incorrect Sensitive feature distributions are not significantly different, as shown in the box plots. A cell is called “Incorrect” if more than 50% of user classifications disagreed with the cell’s predicted classification, based on its MIC and the antibiotic treatment concentration. Notches are drawn showing the median value for each feature, and outliers are shown as spheres. The Bonferroni-corrected p-values were calculated for each pairwise comparison, and the features that were not significantly different are shown with brackets; all the other pairwise comparisons were significantly different with p < 0.0001.

The image features of sensitive and resistant cells that were most often classified correctly were all significantly different (Corrected *t-*test p < 0.001) (Fig. S2). The image features of Correct Sensitive and Incorrect Resistant (truly sensitive) and of Correct Resistant and Incorrect Sensitive (truly resistant) were also significantly different from each other (p < 0.001) (Fig. S2). However, when comparing the features of cells that were most frequently classified incorrectly, there was no significant difference in the mean integrated intensity of the DNA regions (p = 1), the mean standard deviation of intensity of the DNA regions (p = 0.34), and Nucleoid Area Fraction (p = 1) between sensitive and resistant bacteria (Fig. 4), consistent with the images of these cells having features that are too similar to distinguish.

### Images classified correctly and incorrectly clustered separately with distinct feature properties

To understand the cellular phenotypes represented by our image features, a principal component analysis was performed. The principal component analysis allowed us to visualise the phenotypic variance in our image dataset by projecting the feature measurements of each cell into a 2-dimensional space such that images with more similar features would cluster together. In addition, the loading vectors of each feature revealed the magnitude of its contribution to the ciprofloxacin-sensitive and ciprofloxacin-resistant phenotypes.

**Figure 5:**
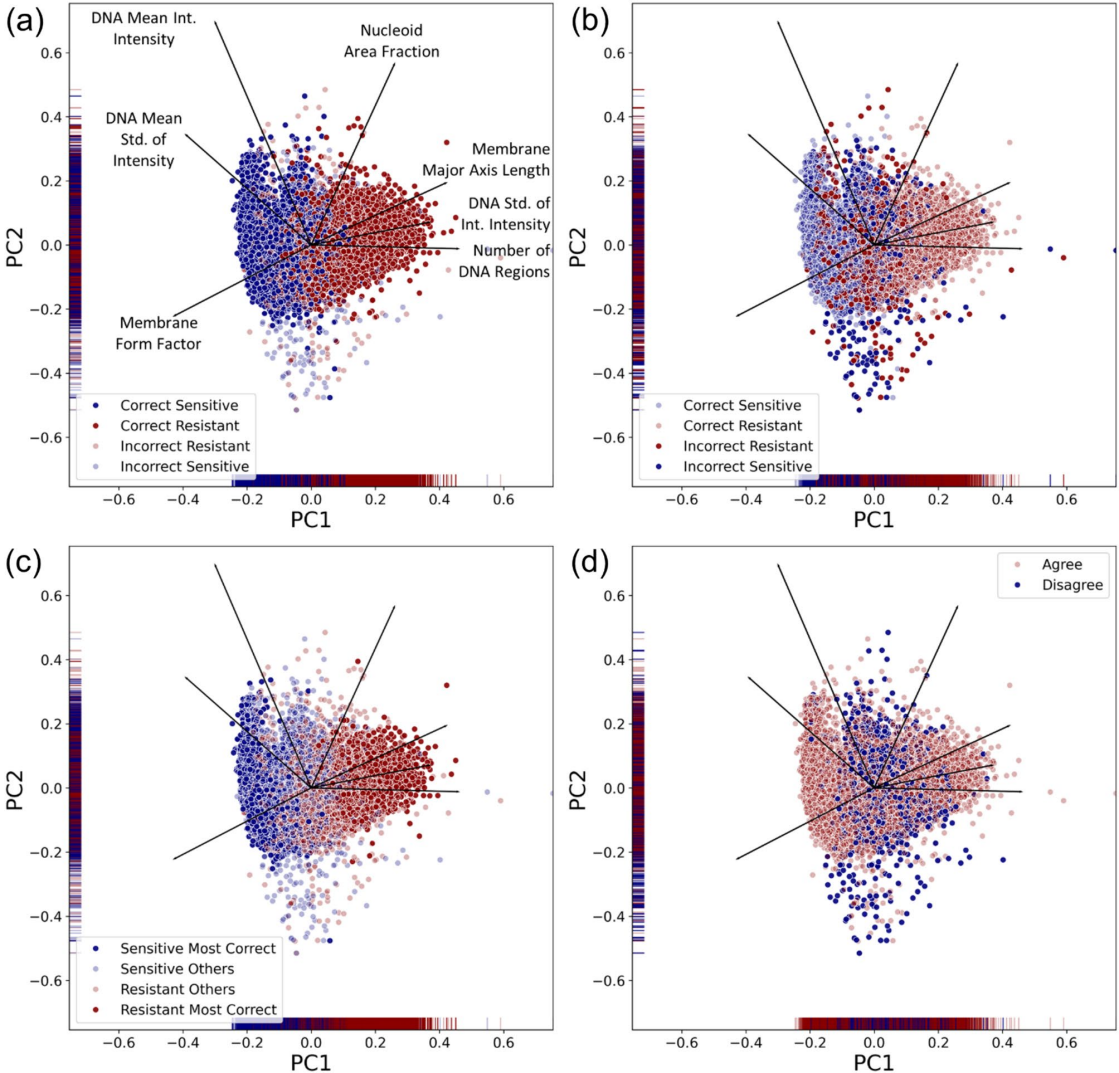
Incorrectly classified images have intermediate phenotypes. (**a**) In a 2-dimensional Principal Component Analysis (PC1, PC2), images of Resistant and Sensitive cells that were classified correctly more than 50% of the time (Correct Sensitive, blue dots and Correct Resistant, red dots) cluster with other members of their class, with some overlap. The feature loadings are shown as black lines. The most influential features for the first Principal Component (PC1) are the Number of DNA Regions and the DNA Standard Deviation (Std.) of Integrated (Int.) Intensity, a measure of the variation in nucleoid region brightness within the cell. (**b**) Images that were classified incorrectly more than 50% of the time cluster near the centre of the principal component plot, or near images of the opposite class, with greater variance in the second principal component than correctly classified images. (**c**) Images that were classified with more than 94% accuracy were called “Most Correct.” The Sensitive Most Correct cells and Resistant Most Correct cells form distinct clusters, indicating that there are certain populations of cells that exhibit characteristic features and are therefore likely to be classified accurately. (**d**) Images where the model and the consensus of users disagree are highlighted in blue. The users are more likely to disagree with the model on images with intermediate features, in the centre, or on images with more atypical features, with greater variance in PC2.

The variation in the first principal component was primarily driven by the number of DNA regions, the standard deviation of the integrated intensity of the DNA regions, and the cell major axis length; the second principal component was driven by the nucleoid area fraction and the mean integrated intensity of the nucleoid (Fig. 5a). Images of sensitive or resistant bacteria that were in the correct class clustered separately, with some overlap (Fig. 5a), while images in the incorrect class clustered in the centre, with greater variation in the second principal component (Fig. 5b). This suggested that images that are frequently classified incorrectly have intermediate phenotypes, with DNA regions and cell lengths that were not clearly demonstrating signs of ciprofloxacin-resistance or sensitivity.

For images where there was greater than 94% accuracy in classification (“Most Correct”; 2438 images), there was a distinct clustering observed with less overlap to the remaining correct images (Fig. 5c). This highlighted that images are more likely to be consistently classified correctly when they exhibit features that distinguish them well from the opposite class.

To compare the classifications of users to our deep learning model, we highlighted images where the user consensus differed from the model’s classification (Fig. 5d). These images are found almost exclusively in the centre of the plot, in between the Most Correct images, and have more variation in the second principal component. This indicates that the model is using more nuanced features from the second principal component to classify images that the users are more likely to misclassify.

### Ciprofloxacin sensitive and resistant cells portrayed different features of importance with respect to correct classification

We investigated which features might be most influential for ciprofloxacin phenotype classification (either by volunteers or a machine learning model) by computing SHAP contributions (SHapley Additive exPlanations)^40^. For the SHAP analysis, a Random Forest classifier was trained to classify images from our dataset as sensitive or resistant using our image feature measurements. An additional feature of normally distributed random numbers was added to determine which features held significance greater than random noise. This model achieved a Mean Absolute Error of 0.15 ± 0.01 on a holdout dataset. The trained Random Forest model was then used to compute SHAP feature contribution scores for each image in the test holdout dataset. The average importance of a feature can be measured by the mean absolute value of the SHAP contribution for all images in the dataset.

**Figure 6:**
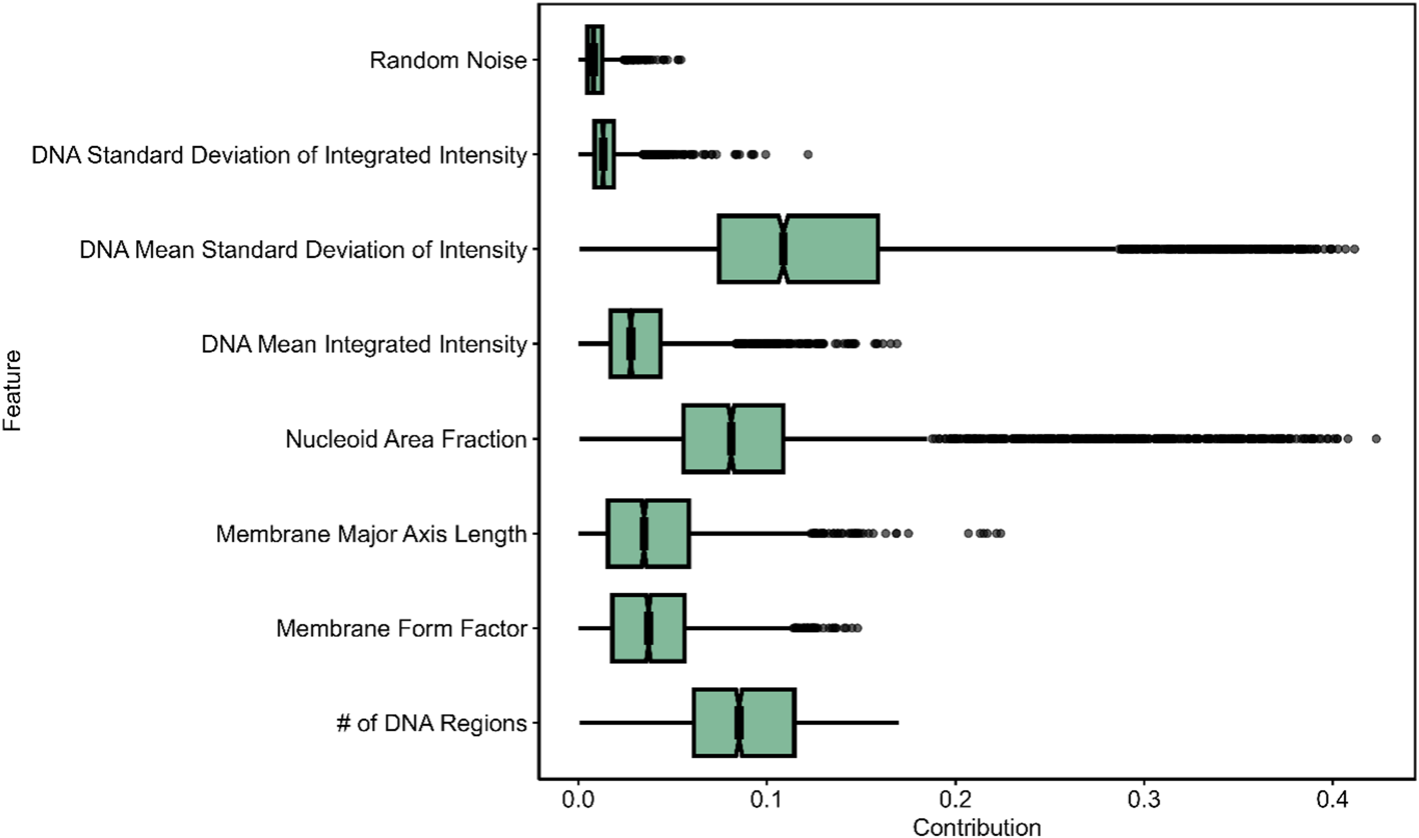
Feature significance for all images measured by SHAP values. The absolute value of the SHAP contribution for each feature is shown on a box plot. In order of their median SHAP contribution, the features are DNA Mean Standard Deviation of Intensity (0.109), Number of DNA Regions (0.085), Nucleoid Area Fraction (0.081), Membrane Form Factor (0.037), Membrane Major Axis Length (0.035), DNA Mean Integrated Intensity (0.028), DNA Standard Deviation of Integrated Intensity (0.013), and Random Noise (0.008). All the features are significantly different from Random Noise (p < 0.0001), and all features are significantly different from each other (p < 0.0001), except Membrane Major Axis Length and Membrane Form Factor (no significance).

Using this approach, and when looking at the entire dataset of sensitive and resistant images, the most important features were the DNA mean standard deviation of intensity (median SHAP = 0.109, *t-*test with Bonferroni correction for multiple comparisons p < 0.0001), number of DNA regions (median SHAP = 0.085, p < 0.0001), and nucleoid area fraction (median SHAP = 0.081, p < 0.0001) (Fig. 6). All of the measured features contributed more to the classification task than the normally distributed random noise (p < 0.0001).

### Different phenotypes developed in resistant *E. coli* treated with high concentrations of ciprofloxacin

In addition to the stark phenotypic differences between ciprofloxacin-treated sensitive and resistant bacteria treated at the same antibiotic concentration, our titration dataset revealed that an *E. coli* strain (EC3) with intermediate resistance (MIC 0.5 mg/L) showed different features when treated at 8-, 16-, and 32-times the MIC (4, 8, and 16 mg/L, respectively) for 30 min compared to 2- and 4-times MIC (1 and 2 mg/L, respectively) (Fig. S3). This matched the trend in classification accuracy for EC3 at these concentrations (Fig. 2b).

## Discussion

The Infection Inspection project showed that misclassifications of 5 tested ciprofloxacin-sensitive and ciprofloxacin-resistant *E. coli* are associated with diversity in the appearance of the bacterial DNA after treatment with ciprofloxacin. Ciprofloxacin is a fluoroquinolone antibiotic that inhibits the enzymes involved in bacterial DNA replication and repair^41^. In sensitive bacteria this can result in the compaction of the DNA and the inability to separate to dividing cells. Whilst our previously reported computer model could achieve a classification accuracy as high as 80%^21^ there remains a degree of classification confusion with respect to certain images, especially near the minimum inhibitory concentration of the strain (Fig. 2b). Infection Inspection volunteers and the computer model had drops in classification accuracy, or increased classification confusion, near the MIC of the strain. This pattern likely reflects intermediate or partial responses to the antibiotic over the 30-min treatment period, which might continue to develop into complete responses or cell death over a longer treatment time. If the classification confusion seen near the MIC in EC1 and EC3 holds for other strains, the concentration at which classification accuracy drops could be used in a rapid AST as an indicator of the MIC of the strain. Interestingly while the model is much more accurate on sensitive cells, users had similar accuracies on sensitive and resistant cells (Fig. 2a). By image feature analysis, we found that images most likely to be classified incorrectly did not show the phenotypic features of correctly classified ciprofloxacin-sensitive or resistant cells, indicating that these bacteria develop ambiguous or intermediate phenotypes.

We used a feature analysis and computed SHAP contribution scores to determine that DNA mean standard deviation of intensity, the nucleoid area fraction, and the number of DNA regions were the most important features when deciding how to classify an image. This means that the degree of DNA compaction and heterogeneity, and the space it occupies within the cell, are the key features that can be used to determine whether an *E. coli* bacterium is responding to ciprofloxacin treatment.

The successful participation of the public with Infection Inspection and the speed at which users classified the images highlights the interest in and value of the public in tackling the problem of antimicrobial resistance. It is clear that citizen science platforms like the Zooniverse provide a valuable resource for recruiting large groups of the public to engage with research^9,10^.

Our project demonstrates the utility of citizen science volunteers in interpreting large biomedical datasets. Biomedical projects are a minority on the Zooniverse platform. A 2019 study showed only 3 biomedical projects of 63 projects surveyed (5%)^19^ were included on the platform; as of November 13, 2023, this fraction remained low, with only 5/100 (5%) of active projects in a biomedical discipline. The Gini coefficient is a measure of inequality that has been used to assess the degree to which many casual volunteers and some super-users contribute to the shared work of Zooniverse projects. On average, biomedical projects were found to have a notably lower average Gini coefficient than astronomy projects, which could be the result of fewer return volunteers, or because biomedical projects more successfully attract many casual contributors^19^. Infection Inspection attracted 3137 volunteers while it was active, with a Gini coefficient of 0.81, higher than the average biomedical project studied. We speculate that our single-step, fast workflow encouraged some users to contribute more classifications than the average biomedical research project.

Despite its successes, the Infection Inspection project had limitations. It relied on voluntary contributions from citizen scientists, which introduced variability in data quality and quantity. We had no information on the users participating in the study, and no quantitative feedback on the impact of our tutorials on informing the public about AMR. The study focused on a single antibiotic and cells obtained from a small number of bacterial strains, limiting the generalizability of findings to other antibiotics and pathogens. Because we utilised images collected for another study, we only had titration data for two of the strains (EC1 and EC3). However, there is potential to use this project design to characterise other bacterial strains, antibiotics, and experimental treatment conditions, such as the dynamic responses of bacterial cells to antibiotics in a time course. Changes in cell size, shape, and DNA morphology have been observed for a range of antibiotics in a number of Gram-positive and Gram-negative species in addition to *E. coli*, such as *Klebsiella pneumoniae*^42^, *Salmonella enterica* serovar Typhimurium^42^, *Staphylococcus aureus*^42^, *Acinetobacter baumanii*^43^, and *Bacillus subtilis*^44^*.* The antibiotic response phenotypes of the most common bacterial pathogens and antibiotics will need to be characterised before a diagnostic test like ours could be brought to the clinic.

Our antimicrobial susceptibility platform is still in early stages of development, but the rich data features captured in this study highlight the potential of a single-cell, imaging-based test for identifying resistant bacterial strains. Although genotype-based assays are rapid at identifying resistance genes with high accuracy (AMRFinder, 98.4%)^45^, the absence of known resistance genes does not always equate to antibiotic sensitivity^4,5^. The accuracy of our approach is currently greater than 88% for sensitive cells, and as more antibiotic response phenotypes are characterized, this should improve. Our method requires a fluorescence microscope and microfluidic chips, but could be implemented as a point-of-care test in a variety of healthcare settings^46^.

Looking ahead, citizen scientists can continue to play a pivotal role in our research and in addressing global health challenges related to antibiotic resistance. Future engagements could involve exploring other bacterial species and antibiotics; improving training materials and guidelines; raising public awareness; and integrating an assessment of the impact of these tools on user education about the scientific topics being studied. Compared to the single classification workflow utilised in this project for user efficiency, other workflow designs could capture more data on the volunteer’s decision-making process. On the project discussion board, some volunteers started discussions about images that appeared to be cells in the process of cell division or images that looked unusual. While the project was not designed to classify images in such detail, it is encouraging to realise that users could be asked to consider stages of cell growth in a future task. Our project, and other researchers working with citizen scientists, can take advantage of this scientific intuition in understanding their datasets.

Comments from volunteers who participated in the Infection Inspection project demonstrate how citizen science methods can be mutually beneficial for research teams and for participants. Several volunteers mentioned that they were motivated to contribute to a project about antibiotic resistance because people close to them had been affected by bacterial infections. One volunteer stated that participating in citizen science projects “gives a sense of pride and usefulness.” Another citizen scientist said that they love this kind of volunteering and that it was “interesting to learn…how bacteria can be acting under the influence of antibiotics.” These comments and others on the project discussion board (https://www.zooniverse.org/projects/conor-feehily/infection-inspection/talk), as well as the enthusiasm with which 5273 volunteers made 1,045,199 classifications, show the interest in biomedical citizen science projects and how such projects can be both educational and meaningful for participants.

In conclusion, the Infection Inspection project exemplifies the potential of citizen science platforms to engage the public in scientific research, enhance the analysis of large datasets, and contribute to our understanding of complex issues like antibiotic resistance. The collaboration between citizen scientists and researchers not only advances scientific methodologies but also fosters a sense of shared responsibility in addressing global health challenges. Despite its limitations, this project has opened doors to further exploration and collaboration, highlighting the promising role of citizen science in the future of biomedical research and public health.

### Supplementary Information

Supplementary Information.

## Data Availability

The raw data images used to build this project are available from the Oxford University Research Archive: https://ora.ox.ac.uk/objects/uuid:12153432-e8b3-4398-a395-abfb980bd84e. The individual segmented single cell images and classification metadata are available at: 10.5281/zenodo.10301352.
