# Supplementary material for: Infection Inspection: using the power of citizen science for image-based prediction of antibiotic resistance in Escherichia coli treated with ciprofloxacin
Source: Sci Rep. 2024 Aug 22;14:19543. doi: 10.1038/s41598-024-69341-3 (PMC11341553; doi:10.1038/s41598-024-69341-3)

Supplementary information for "**Infection Inspection: Using the power of citizen science for image-based prediction of antibiotic resistance in *Escherichia coli* treated with ciprofloxacin**"

Contents:

- **Figure S1.** Accuracy of users based on engagement.
- **Figure S2.** The definition and distributions of the seven measurements used for image feature analysis.
- **Figure S3.** Resistant *E. coli* treated with high concentrations of ciprofloxacin cluster together.
- **Supplementary Methods:** Infection Inspection Tutorial
- **Supplementary Methods:** Infection Inspection Field Guide

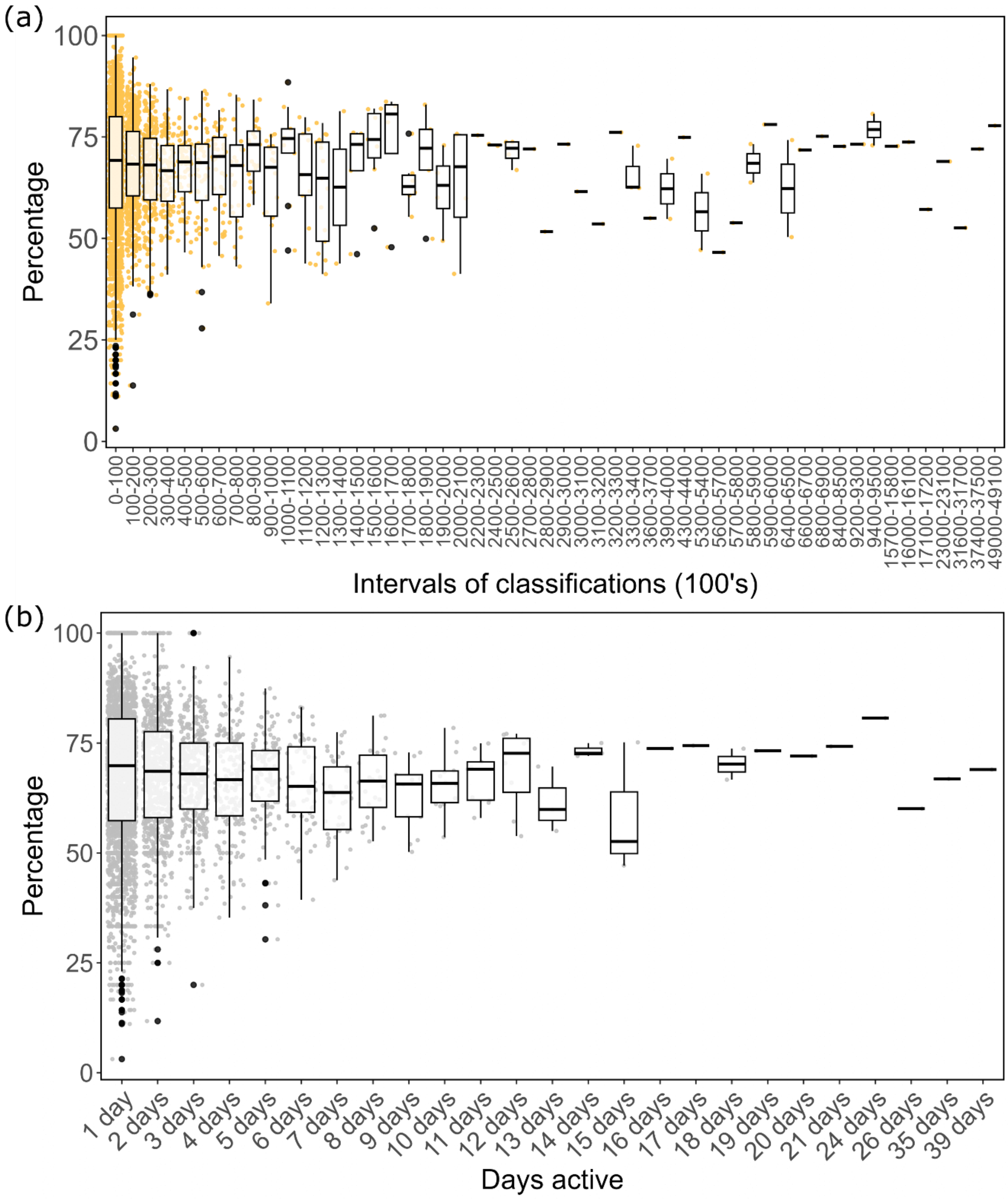

Figure S1. Accuracy of users based on engagement. Each boxplot represents the median image classification accuracy for users based on: (a) the total number of images they classified, or (b) total numbers of days they accessed the project. Boxplots highlight the middle 50% of data (IQR) with the median image classification accuracy for users shown in the central line.

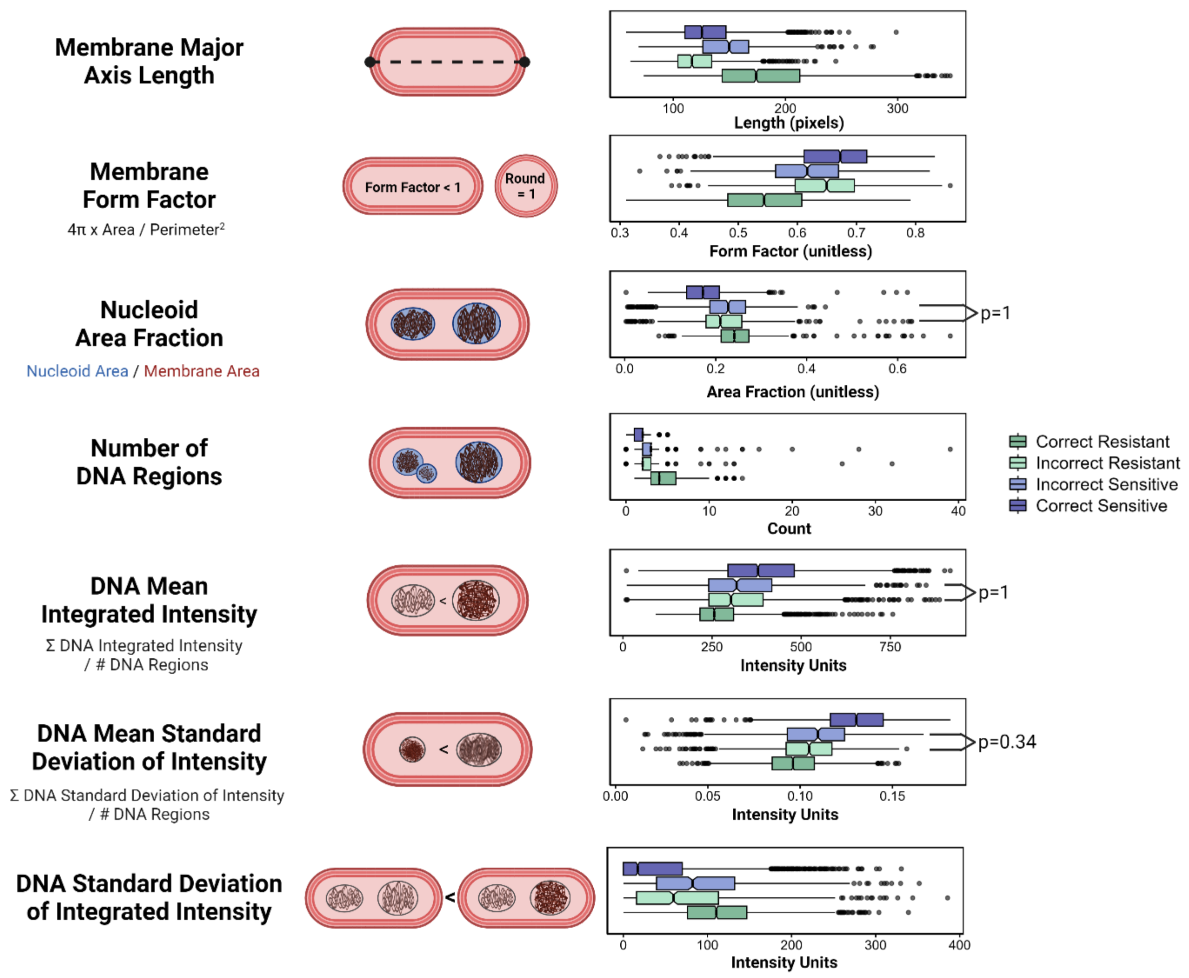

Figure S2. The definition and distributions of the seven measurements used for image feature analysis. Seven measurements were chosen for their potential to reflect responses to ciprofloxacin treatment. The features, with illustrative diagrams, are shown on the left; box plots of the feature distributions for all cells are shown on the right, coloured by whether the cell was Sensitive or Resistant and whether they were most often classified Correctly or Incorrectly. Notches indicate the median value and outliers are plotted as black dots. The Membrane Major Axis Length and the Membrane Form Factor measure cell size and cell shape, respectively. The Form Factor of a perfectly round object is equal to 1, so most bacilli will have form factors <1. The Nucleoid Area Fraction is a measurement of DNA compaction, DNA size, and cell size. The Number of DNA Regions detected by CellProfiler also reflects DNA compaction and cell cycle stage. Other measurements of the nucleoid, such as the Mean Integrated Intensity, Mean Standard Deviation of Intensity, and Standard Deviation of the Integrated Intensity of DNA regions, measure the changes in DNA heterogeneity and compaction as *E. coli* respond to ciprofloxacin. These measurements also capture the variations in nucleoid morphology that can be seen within the same cell. For each feature, pairwise t-tests were performed for the Correct Resistant, Incorrect Resistant, Incorrect Sensitive, and Correct Resistant distributions. The p-value with Bonferroni correction for multiple comparisons is <0.0001 except where labelled.

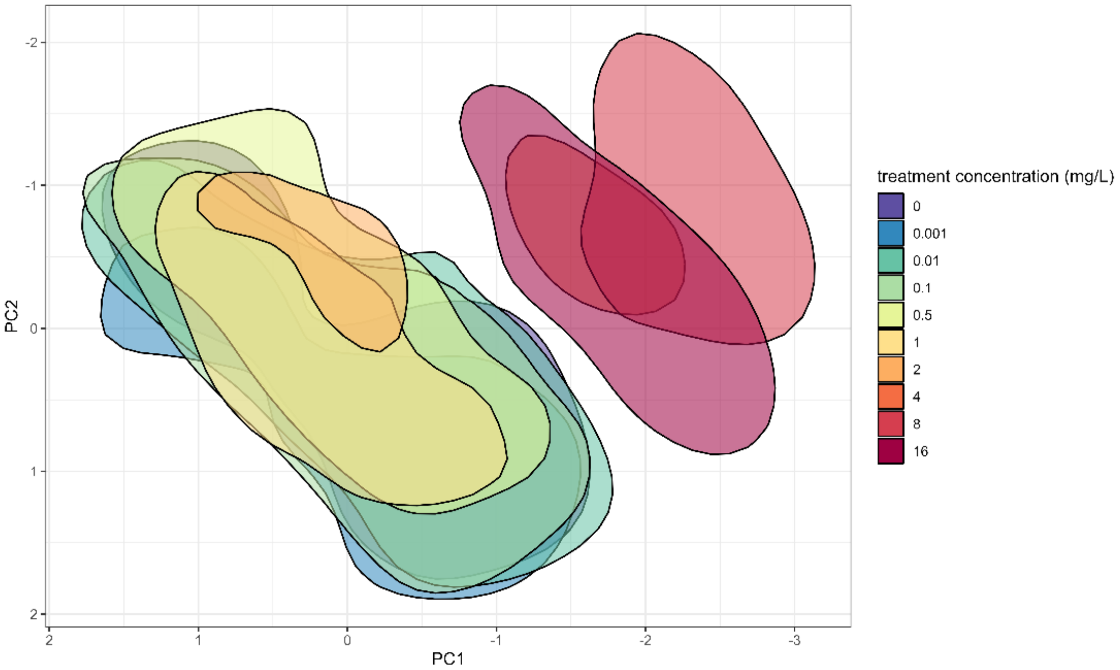

Figure S3. Resistant *E. coli* treated with high concentrations of ciprofloxacin cluster together. This ciprofloxacin-resistant clinical isolate (EC3; MIC = 0.5 mg/L) was treated at varying concentrations of ciprofloxacin for 30 minutes. This principal component analysis shows that features of cells treated at extremely high concentrations of ciprofloxacin (8-, 16-, and 32-times MIC; 4, 8, and 16 mg/L) form a separate cluster from those treated at lower concentrations, even when those concentrations are above the MIC. Geometric shapes are plotted with different colours to show the regions with point density above 0.07.

Supplementary Methods: Infection Inspection Tutorial

(1) What's it all about?

We want you to tell us if you think the bacteria that you see is resistant or sensitive to the antibiotic ciprofloxacin. In this workflow you will see images of *Escherichia coli* (*E. coli*) that we took on a laboratory microscope.

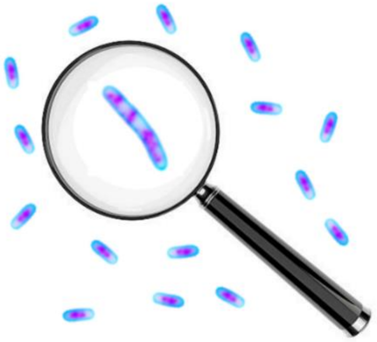

Read the Field Guide tab for some helpful information about identifying antibiotic resistance in our bacteria before starting your classification task.

(2) What does resistance look like?

When a bacterium is resistant to the effects of ciprofloxacin, its DNA will be more spread out (diffuse), throughout the bacterium, and there might be multiple copies of the DNA.
In all of the images you will see, the bacterial DNA will be shown in green and the cell membrane will be shown in red.

Image colour can be inverted to help differentiate the DNA and cell membrane.

(right) Examples of a resistant *E.coli*

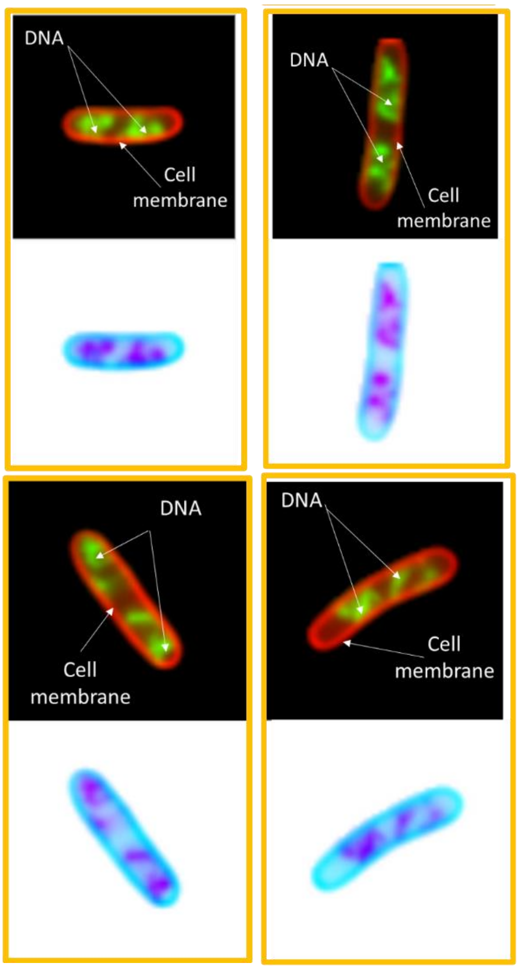

(3) What does sensitivity look like?

When a bacterium is sensitive to ciprofloxacin, the DNA will break when the cell tries to make more DNA in order to divide. In a sensitive bacterium, the DNA will collapse into a smaller, denser ball.

(right) Examples of a sensitive *E.coli*

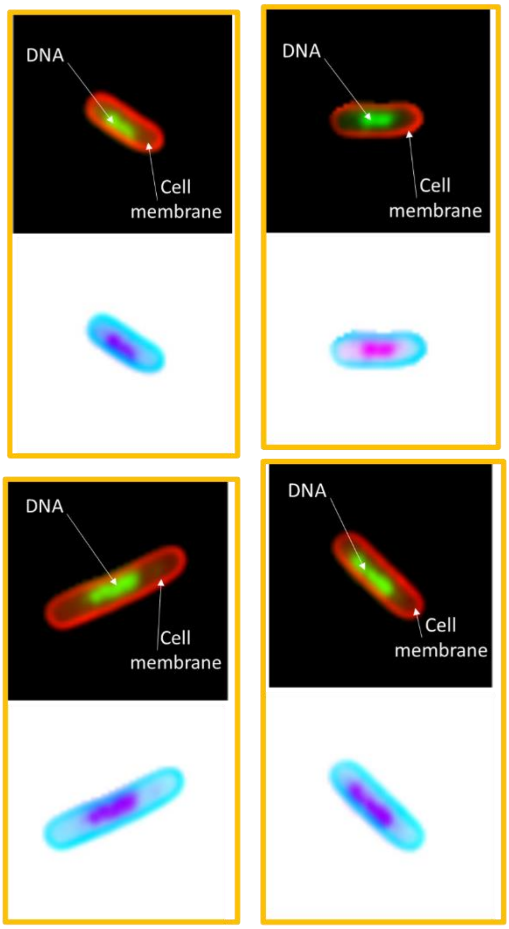

(4) Be careful...

All of these images are just a snapshot of the cell's response. The images are captured after 30 minutes of antibiotic treatment.

In some cases, there may be sensitive cells where the DNA is not yet completely condensed. In the example below, you can see a sensitive cell that still has two distinct clumps of DNA, but, the DNA in each clump is more compact than in the resistant example.

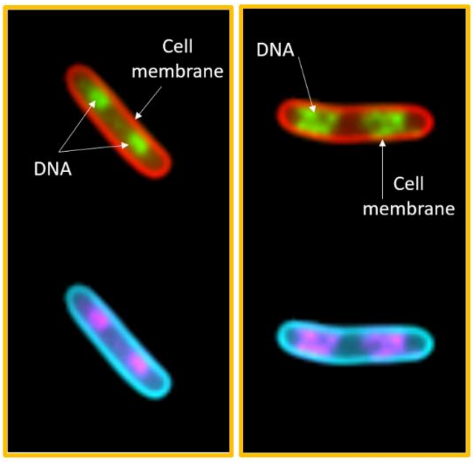

Sensitive Resistant

(5) Images gone wrong

We capture thousands of cells in one field of view on the microscope and use image analysis software to generate the single images of the cell.

In some cases, this processing step can go wrong and generate an image that is blurry, or not even a cell. You might encounter some of these from time to time, like in the examples below. If the cell membrane (red) looks weird but you can still see the DNA (green) signal well enough to classify the cell as Sensitive or Resistant, that's the best thing to do. Otherwise, if the image is impossible to classify, just select image processing error.

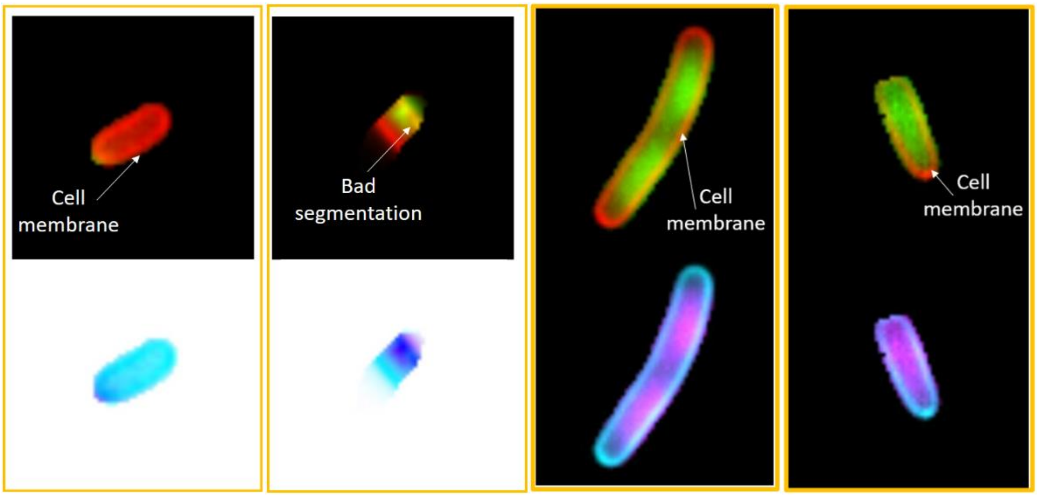

(6) Let's go!

Please select from one of the 3 options, submit your answer, and then move on to another image.

All selections are autosaved when you click submit.

If you are unsure whether a bacterium looks like it is resistant or sensitive to ciprofloxacin, try to give a best guess based on how compact or not the DNA looks.

Supplementary Methods: Infection Inspection Field Guide

1. How to Identify Antibiotic Resistance

Antibiotics work by blocking essential parts of the bacterial life cycle. For example, ciprofloxacin blocks an enzyme that the bacteria needs to untangle and repair supercoiling of its DNA. Without this, the DNA will break when the bacteria try to replicate and divide. This usually results in the death of the bacteria.

In this workflow you will see images of *Escherichia coli* that we took on a laboratory microscope.

Escherichia coli, or *E. coli* for short, is a group of bacteria. Many strains of *E. coli* are harmless and can be found in the environment and in the digestive tract of humans and animals, but other strains are pathogenic, meaning they cause infections that make people sick.

When someone is sick with an infection, the clinicians treating them need to give them an antibiotic that can stop the infection. When bacteria are sensitive to an antibiotic, this means that the antibiotic can either kill the bacteria or prevent how the bacteria normally operates.

We want to learn how to identify antibiotic-resistant bacteria in our images so that we can develop a faster test to detect them. We hope this will help doctors treat patients with bacterial infections. Before taking these pictures, we labelled parts of the bacteria so that we can see how they respond to treatment with the antibiotic.

The plasma membrane was stained with Nile Red and you will see it as the red structure lining the bacteria in the images.

The DNA or nucleoid was stained with DAPI and you will see it as the green structure inside the bacteria.

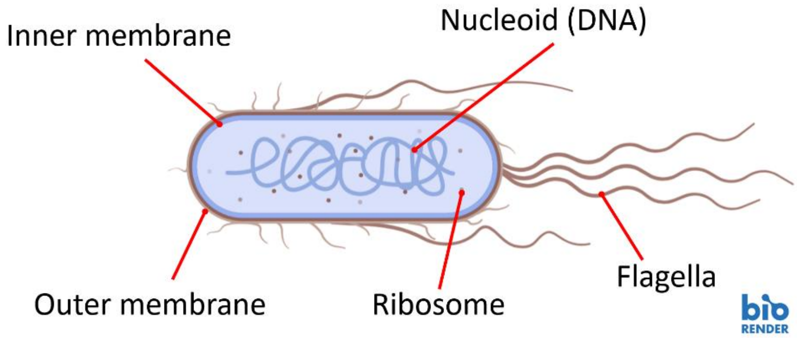

This is a drawing of the parts of an *E. coli* bacterium. In our images, we decided to label the plasma membrane and the DNA.

Examples of a resistant *E. coli*

When a bacterium is resistant to the effects of ciprofloxacin, its DNA will look more like the DNA in an untreated bacterium. The DNA will be more spread out, or diffuse, throughout the cell, and there might be two copies of the nucleoid.

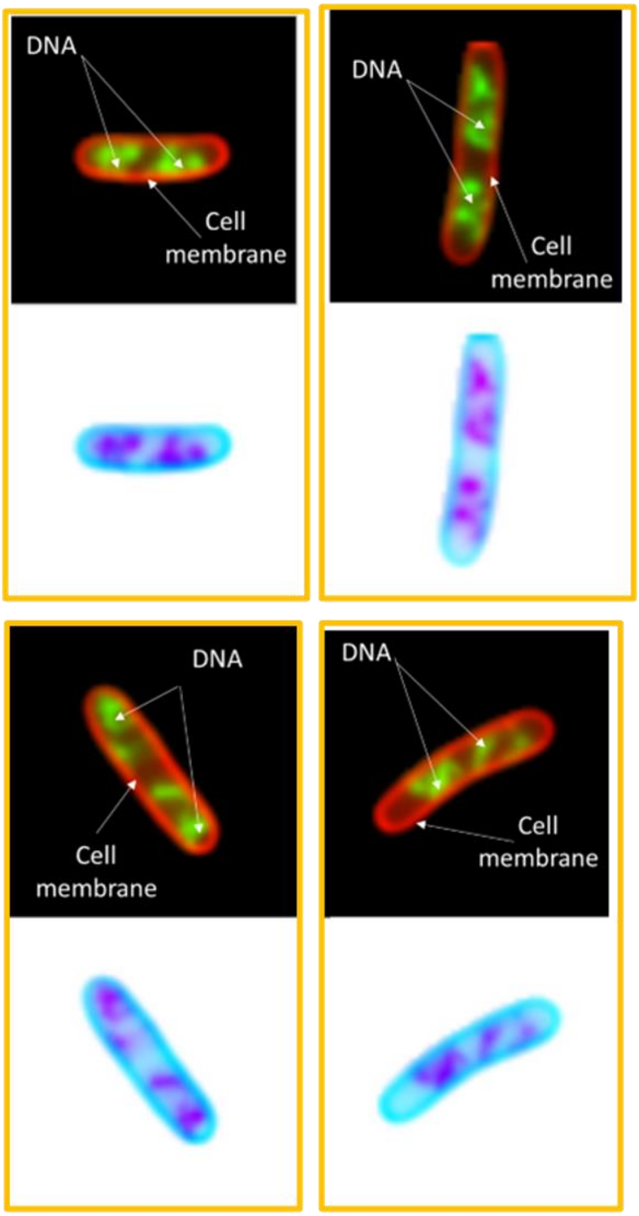

Examples of a susceptible *E. coli*

When a bacterium is sensitive to ciprofloxacin, the DNA will get broken when the cell tries to make more DNA in order to divide. In a susceptible cell, the DNA will collapse into a smaller, denser ball.

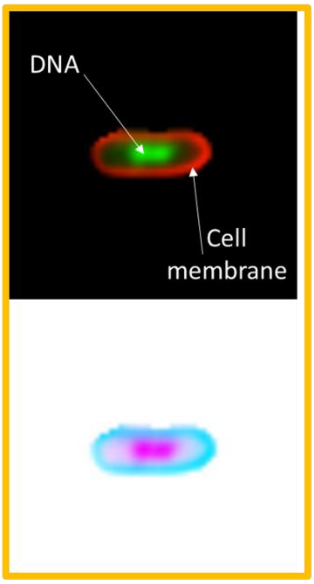

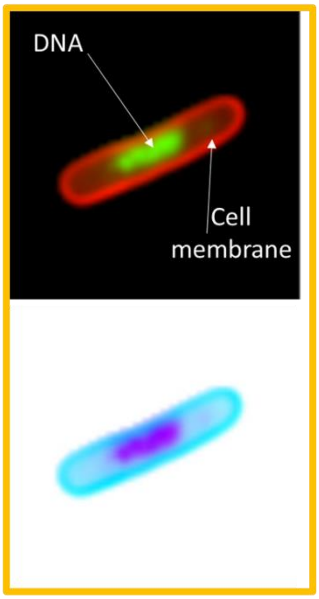

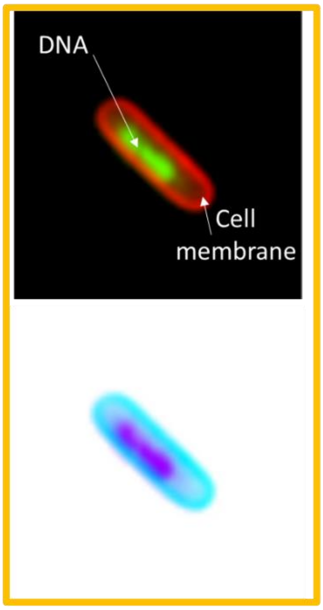

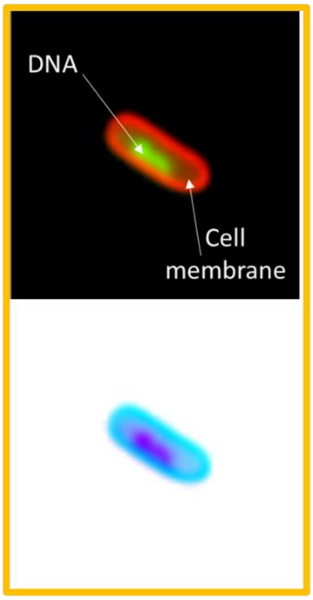

- Keep these principles in mind as you classify our images. If you are unsure whether a given cell is susceptible or resistant to antibiotics, make your best guess based on these principles and your intuition.

2. Resistant examples

If the bacterium is resistant to this antibiotic, the DNA will be more spread out across the cell.

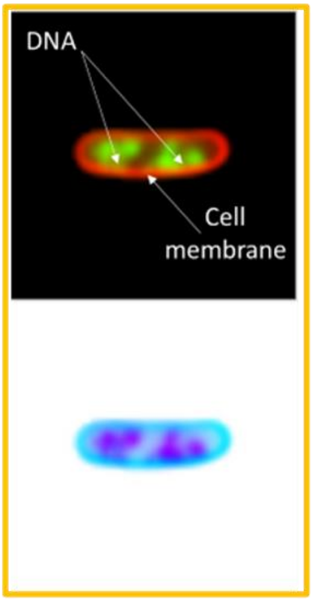

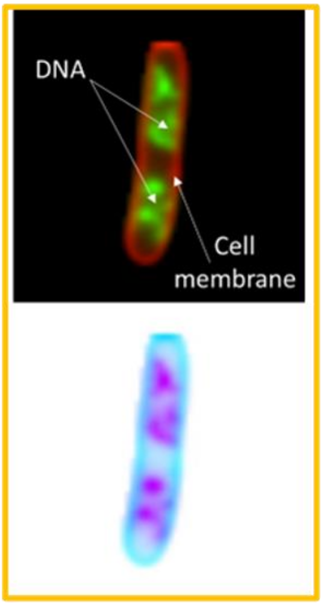

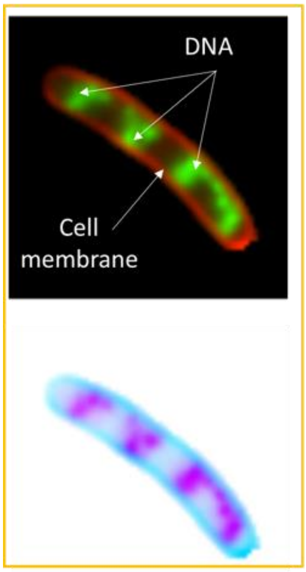

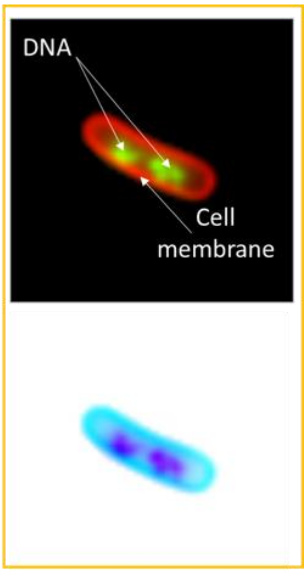

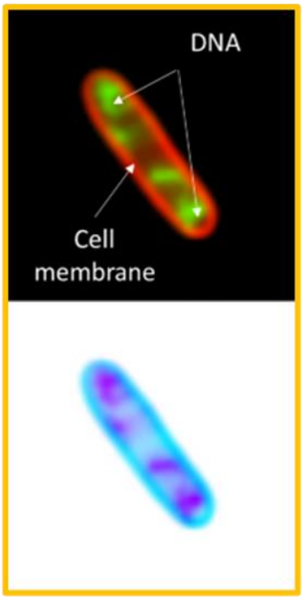

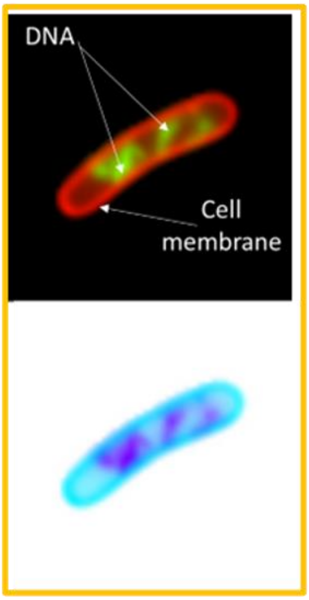

3. Sensitive examples

When a bacterium is sensitive to this antibiotic, the DNA will be more compact in the cell. In most cases it will be just a dot in the centre of the cell.

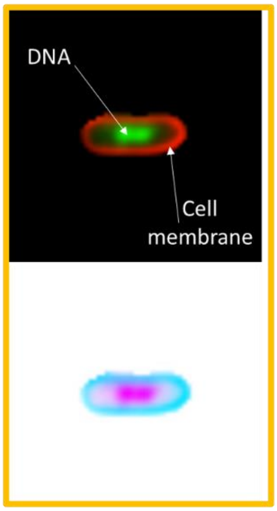

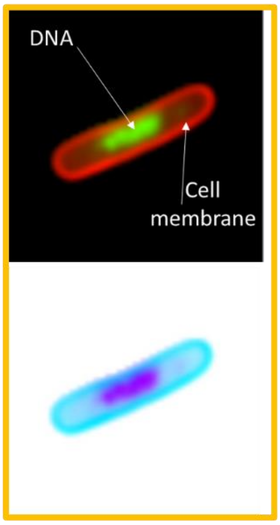

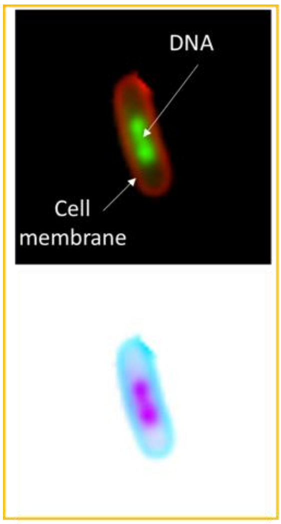

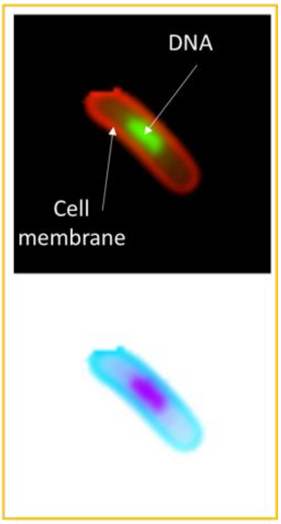

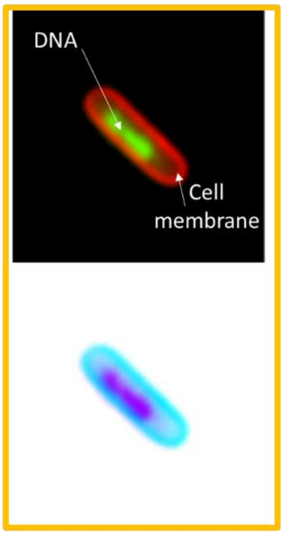

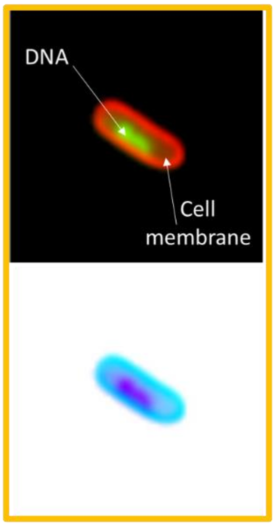

4. Image Processing Errors

From time to time, you may encounter a picture that is just a few coloured squares, like in the image above. This is an artifact from the image processing pipeline. You can label these images as "Imaging error."

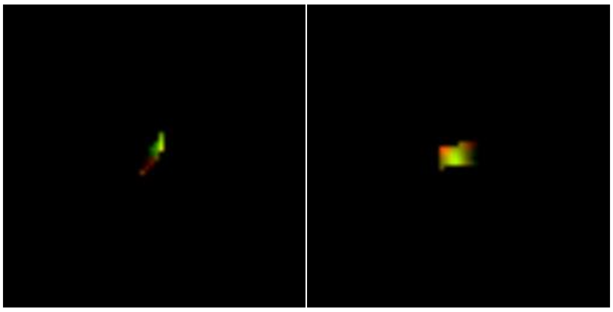

Sometimes there is a problem with the imaging and staining of the DNA and you might see images like this one. It looks like the DNA is spotted all over the cell, but this is an artifact. **You can classify a cell like this as an "Imaging error."**

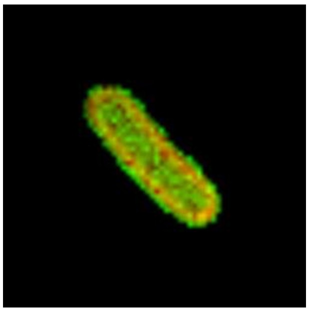

Here is another example of what might happen if something goes wrong with the imaging and staining of the DNA. It can look like an empty cell or look like there are two spots of DNA signal at the ends of the cell and on top of the membrane. This is an artifact of the image processing. **You can classify a cell like this as an "Imaging error."**

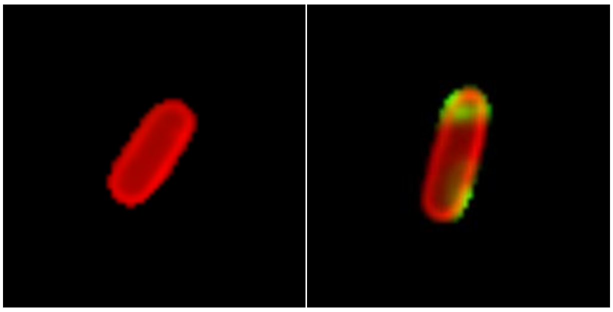

We use the cell membrane image to trace an outline of the cell and segment it, or remove it from the background of the image. Sometimes this process is imperfect, and the cell membrane can look pixelated or parts of the membrane can be cut off. As long as you can see enough of the DNA to make a classification, it's best to choose "Resistant" or "Sensitive." If the segmentation is so bad that you cannot make a classification, it's alright to choose "Image Processing Error." In the image below, it would be possible to still classify the cell based on the appearance of the DNA.

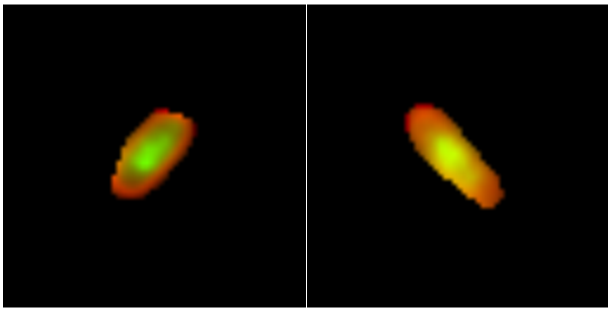

5. Unusual looking cells

Sometimes the staining and imaging might produce bacteria that look unusual. There are numerous reasons for this, but if you see a bacterium that looks very different from the others you've seen, then try to make the best guess that you can.

In this image, the cell membrane (red) has been traced imperfectly during the image processing. You can't see much of the cell membrane, but you can still see the DNA (green) enough to judge its shape and whether it has responded to the antibiotics. **You should still classify a cell like this by looking at the DNA.**

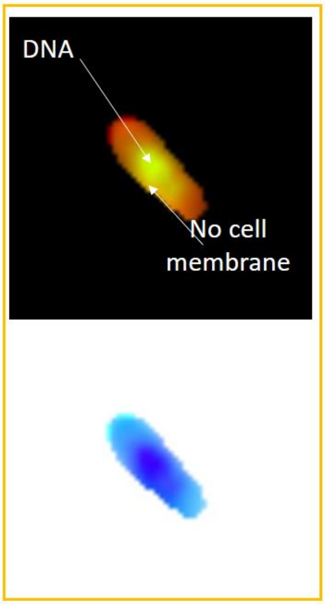

In this image, it looks like the DNA (green) is outside of the cell and on top of the membrane (red). The DNA is always inside of the membrane in a living bacterium. In this case, the image processing likely misaligned the data from the DNA image and the membrane image. **You should still be able to classify a cell like this by looking at the structure of the DNA.**

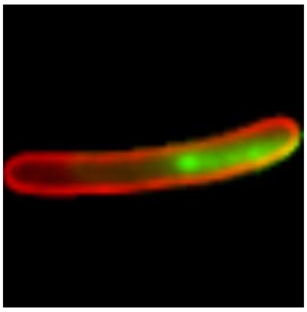

The overall length of the cell does not matter so much in this task. Like the image above and in the image below, some cells might appear very long. In the example below however, the DNA is quite compact, and so you would classify this cell as sensitive.

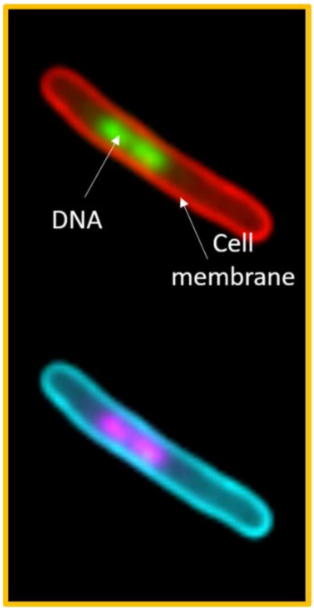

Supplement: Supplementary file 1 — Supplementary Information. [file 41598_2024_69341_MOESM1_ESM.pdf]
